# Interdisciplinary dialogue for education, collaboration, and innovation: Intelligent Biology and Medicine in and beyond 2013

**DOI:** 10.1186/1471-2164-14-S8-S1

**Published:** 2013-12-09

**Authors:** Bing Zhang, Yufei Huang, Jason E McDermott, Rebecca H Posey, Hua Xu, Zhongming Zhao

**Affiliations:** 1 Department of Biomedical Informatics, Vanderbilt University School of Medicine, Nashville, TN 37232, USA; 2 Department of Cancer Biology, Vanderbilt University School of Medicine, Nashville, TN 37232, USA; 3 Center for Quantitative Sciences, Vanderbilt University, Nashville, TN 37232, USA; 4 Department of Electrical and Computer Engineering, The University of Texas at San Antonio, San Antonio, TX 78249, USA; 5 Computational Biology and Bioinformatics Group, Pacific Northwest National Laboratory, Richland, WA 99352, USA; 6 School of Biomedical Informatics, The University of Texas Health Science Center, Houston, TX 77030, USA; 7 Department of Psychiatry, Vanderbilt University School of Medicine, Nashville, TN 37212, USA

## Abstract

The 2013 International Conference on Intelligent Biology and Medicine (ICIBM 2013) was held on August 11-13, 2013 in Nashville, Tennessee, USA. The conference included six scientific sessions, two tutorial sessions, one workshop, two poster sessions, and four keynote presentations that covered cutting-edge research topics in bioinformatics, systems biology, computational medicine, and intelligent computing. Here, we present a summary of the conference and an editorial report of the supplements to *BMC Genomics* and *BMC Systems Biology* that include 19 research papers selected from ICIBM 2013.

## Introduction

Built upon the success of last year's conference [1-4], the 2013 International Conference on Intelligent Biology and Medicine (ICIBM 2013) was held on August 11-13, 2013 in Nashville, Tennessee, USA. The primary goal of the conference remains to foster interdisciplinary and multidisciplinary research and to provide education and training opportunities to students and junior investigators who are interested in bioinformatics, systems biology, or intelligent computing. The conference brought together more than 110 participants with diverse backgrounds spanning biology, medicine, computer science, bioengineering, statistics, and mathematics, among others.

We received 65 manuscript and 37 abstract submissions. Compared to last year, we continue to have steady submissions on topic areas including biological network analysis, network medicine, and next-generation sequencing (NGS) data analysis. An emerging research area at ICIBM is proteomics-based research and applications. Thanks to grant support from the National Science Foundation, we were able to provide 21 travel awards to trainees from 19 universities across both the USA and international institutions. The travel awards were selected by the Award Committee from a substantial number of outstanding manuscripts and abstracts that spanned the wide variety of research subjects. In the following section, we present a summary of the scientific program of the conference and an editorial report of the supplements to *BMC Genomics* and *BMC Systems Biology*.

## ICIBM 2013 scientific program

The scientific program included four keynote speakers who are world renowned leaders in biomedical informatics, pharmacogenomics, bioinformatics, and systems biology, six scientific sessions, two poster sessions, two tutorials, and one workshop. Here, we briefly review the keynote speakers' lectures followed by the workshop, tutorials, and regular scientific sessions.

Four keynote speakers presented their pioneering research and shared their perspectives of relevant research fields. These speakers were Dr. Lucila Ohno-Machado from the University of California, San Diego, Dr. Dan M. Roden from Vanderbilt University, Dr. A. Keith Dunker from Indiana University, and Dr. Yixue Li from the Chinese Academy of Sciences.

"*Sharing clinical and genomic data for research: Is it simply a matter of trust?*" Dr. Ohno-Machado presented different models for sharing clinical and genomic data for research, which are designed to accommodate highly diverse policies. She also discussed how her group is currently implementing these models in several projects, such as the University of California Research eXchange initiative. Dr. Ohno-Machado is the Associate Dean for Informatics and Technology at the School of Medicine, University of California, San Diego, the founding Chief of the Division of Biomedical Informatics, and a Professor of Medicine. She is an elected fellow of the American Institute for Medical and Biological Engineering, the American College of Medical Informatics, and the American Society for Clinical Investigation. She is the Editor-In-Chief of the Journal of the American Medical Informatics Association. Her research focuses on predictive modeling, particularly including the evaluation of individualized probabilistic estimates for risk assessment and prognosis.

"*Genetic variation modulating drug response: discovery and implementation*" Dr. Roden introduced BioVU, a resource that links DNA extracted from clinically-obtained blood samples to their de-identified electronic medical record (EMR). BioVU not only enables the discovery of new genomic variants associated with specific clinical phenotypes, but also new phenotypes associated with specific genotypes (i.e. genetic pleiotropy) in an approach Dr. Roden and his team termed "phenome-wide association study" (PheWAS). Dr. Roden also presented the Vanderbilt PREDICT (Pharmacogenomic Resource for Enhanced Decisions in Care and Treatment) program that empowers patients and doctors with the genetic information needed to predict and help prevent adverse side effects of drugs. Dr. Roden served as the Director of the Vanderbilt Arrhythmia Service, the director of the Division of Clinical Pharmacology (1992-2004), and in 2006 was named the Assistant Vice-Chancellor for Personalized Medicine. Dr. Roden has been elected to membership in the American Society for Clinical Investigation and the Association of American Physicians, and he is a fellow of the American Association for the Advancement of Science.

"*An intrinsically disordered protein Swiss-Knife-like toolkit for signaling diversification*" Dr. A. Keith Dunker provided a comprehensive review of intrinsically disordered proteins (IDPs) and their critical role as a multifaceted, Swiss-Knife-like toolkit that enables swift (on an evolutionary time scale) diversification of cell signaling to facilitate the development of metazoans and their rapid evolution. Dr. Dunker is a Professor of Biochemistry and Molecular Biology at Indiana University, where he launched the Center for Computational Biology and Bioinformatics and served as its Director. He is best known for his research in understanding IDPs using bioinformatics approaches and laboratory experiments. He and his collaborators were the first to consider these proteins as a distinct class with important biological functions.

"*Genome sequences of wild and domestic bactrian camels*" Dr. Yixue Li presented draft genome sequences from both a wild and a domestic *Bactrian *camel. The study by Dr. Li and his team reveals the evolutionary history of camels and provides insights on the genetic basis of camels' remarkable salt tolerance and unusual immune system. Dr. Li is the Director of the Shanghai Center for Bioinformation Technology, Vice Director and Professor of the Key Laboratory of Systems Biology at Shanghai Institutes for Biological Sciences, Chinese Academy of Sciences. Dr. Li's research interests include bioinformatics, systems biology, and computational biology.

ICIBM 2013 included one workshop and two tutorials for educational purposes, all of which were much appreciated by the conference participants.

"*Workshop on Next-Generation Sequencing*" This workshop was organized by Dr. Kun Huang from The Ohio State University and Dr. Dongxiao Zhu from Wayne State University. The workshop brought together active researchers in the NGS field and provided an opportunity for them to introduce cutting edge technologies and novel computational methodologies, discuss challenges and opportunities, and interact with the attendees. This workshop had three sessions and nine talks. A majority of the talks introduced new methods for NGS data analysis, including genotype calling, identifying spontaneous mutations in bacteria, metagenomic mining, peak-calling in ChIP-Seq experiments, network-based detection of cancer driver genes, and analysis of allele specific expression. Others presented interesting applications of NGS technologies to studies in autism spectrum disorder, cancer, and pharmacogenomics.

"*Tutorial I: Introduction to Proteome Informatics*" This tutorial was organized by Dr. David L. Tabb from Vanderbilt University, and it had four instructors. The workshop introduced major elements of the protein identification and quantitation pipelines and describes a strategy for proteogenomic experiments with both RNA-Seq and proteomic data. The workshop provided a useful overview of proteome informatics for computer scientists, bioinformaticians, and statisticians who have not previously worked with proteomics data sets.

"*Tutorial II: Pathway and Network Analysis Tutorial*" This tutorial was provided by Dr. Alexander Pico from the Gladstone Institutes and Dr. Jing Wang from Vanderbilt University. Dr. Pico provided a general introduction to WikiPathways, a collaborative platform for building, curating, and distributing biological pathway knowledge for the research community. He also provided a brief introduction to the powerful network visualization tool Cytoscape. Dr. Wang introduced NetGestalt, a novel web-based data integration framework that allows simultaneous presentation of large-scale experimental and annotation data from various sources in the context of biological networks to facilitate data visualization, analysis, and interpretation.

ICIBM 2013 had six regular scientific sessions for researchers to showcase their original works in the areas of bioinformatics, systems biology, medical informatics, and intelligent computing. The presenters were chosen through a rigorous review process, and their work stood out among the submissions as novel and significant. These sessions were:

Session I: Next Generation Sequencing (NGS): Analysis and Tools

Session II: Network Analysis

Session III: Genomics

Session IV: Systems Biology

Session V: Computational Medicine

Session VI: Intelligent Computing

The details of each session, including session chairs, speakers, and the title and abstract of each talk, are available online [5] and in the conference program book. Here, we provide an editorial report of the supplements to BMC Genomics and BMC Systems Biology that include 19 research papers selected from 65 manuscripts submitted to ICIBM 2013. Each manuscript was reviewed by at least two reviewers (most by three reviewers) and went through two rounds of reviews. Among the 19 selected papers, 8 are devoted to network analysis methods and their applications to disease studies. Four papers describe new development or careful evaluation of methods for NGS data analysis. Two papers employ proteomic or proteogenomic approaches in human cancer studies. The other papers cover a diverse range of topics.

## Network analysis methods and applications

A large proportion of papers focused on network analysis methods and their application to human disease studies. Udyavar et al. [6] applied the weighted gene co-expression network analysis in a lung cancer study and uncovered a signature of signaling hubs closely associated with the small cell lung cancer (SCLC) phenotype. Among the identified hubs, tyrosine kinase SYK emerged as an unsuspected SCLC oncogenic driver and potential therapeutic target. Yu et al. [7] integrated co-expression and the protein interactome to identify network modules of human diseases. The method outperformed the traditional differential expression approach. Budd et al. [8] used a network-based approach that determines the sum node degree for all experimentally verified microRNA targets in order to identify potential regulators of prostate cancer initiation, progression, and metastasis. Shi et al. [9] developed a two-step approach for gene regulatory network identification, featuring an integrated method to identify modularized regulatory structures and subsequently refine their target genes. Ma et al. [10] developed a tool for modeling and visualizing the relationship between different groups of compounds that share similar differential gene expression signatures, termed "Mode of Actions," regarding their therapeutic effect. They then applied the tool to a breast cancer study. Wu et al. [11] built a weighted disease and drug heterogeneous network based on known disease-gene and drug-target relationships and then clustered the network to identify modules and infer putative drug repositioning candidates. Liu et al. [12] proposed the use of graph-based Laplacian regularized logistic regression to integrate biological networks into disease classification and pathway association problems. The algorithm outperformed elastic net and lasso in the simulation studies. The utility of the algorithm was also validated through its ability in reliably differentiating breast cancer subtypes using a breast cancer dataset from The Cancer Genome Atlas (TCGA) consortium. Finally, Jiang et al. [13] proposed a comprehensive framework at the network level to integrate single nucleotide polymorphism (SNP) annotation, target gene assignment, Gene Ontology classification, pathway enrichment analysis, and regulatory network reconstruction to illustrate the molecular functions of prostate cancer-associated SNPs.

## NGS data analysis methods and applications

Several papers presented new methods or thorough evaluations of existing methods for the analysis of data derived from metagenomic sequencing, ChIP-Seq, or RNA-Seq. Srinivasan et al. [14] developed an alignment-free n-gram-based method named MetaID that can accurately identify microorganisms at the strain level and estimate the abundance of each organism in a sample given a metagenomic sequencing dataset. Liu et al. [15] developed a novel quantitative method for comparing two biological ChIP-Seq samples, called QChIPat. Their method has several advantages. First, it considers a control (or input) experiment; second, it incorporates a nonparametric empirical Bayes correction normalization; moreover, it provides the binding pattern information among different enriched regions. Guo et al. [16] designed a comprehensive experiment to evaluate six read count-based RNA-Seq analysis methods (DESeq, DEGseq, edgeR, NBPSeq, TSPM and baySeq) using both real and simulated data. They found the six methods produce similar fold changes and reasonable overlapping of differentially expressed genes. However, all six methods suffered from over-sensitivity. Compared to other methods, edgeR achieved a better balance between speed and accuracy. Liu et al. [17] analyzed RNA-Seq data from kidney renal clear cell carcinoma at both gene- and isoform-levels in an attempt to uncover cancer-stage-dependent expression signatures. They found that isoform expression profiling provides unique and important information that cannot be detected by gene expression profiles. Furthermore, they showed combining gene and isoform expression signatures helps identify advanced stage cancers, predict clinical outcome, and present a comprehensive view of cancer development and progression.

## Proteomics in cancer research

Molecular cancer research has been dominated by genomic technologies during the last decade. With recent advancements in proteomics technologies, proteomics and integrative proteogenomics now play an increasingly important role in this field. Sun et al. [18] created the database CanProFu that comprehensively annotates fusion peptides formed by exon-exon linkage between these pairing genes. They applied the database to mass spectrometry datasets of 40 human non-small cell lung cancer (NSCLC) samples and 39 normal lung samples and identified 11 NSCLC-specific gene fusion events. Zhang et al. [19] presented a peptidomics approach to search for novel alternative splicing isoforms in clinical proteomics. Their results showed that the approach has significant potential in enabling the discovery of new types of high-quality alternative splicing isoform biomarkers. Proteomics datasets have also been used to confirm a SCLC gene expression signature identified from microarray data [6].

Other papers in these supplements cover a diverse range of topics. Dai et al. [20] comprehensively analyzed the sequence origin of Pldi-Ak158810 loci, which originated from the inter-genic regions in mice after the divergence of mice and rats. They found that various factors, including rearrangement and transposable elements, contributed to the formation of the sequence. To address the multiple-test correction problem in expression quantitative trail loci (eQTL) studies, Chakraborty et al. [21] developed an approach that takes advantage of an empirical Bayes method and local false discovery rate (lfdr) calculation. Their method better controls the false positive rate compared to traditional methods. Tyaga et al. [22] developed a 3D QSAR model that allows researchers to correlate the structural features of thiosemicarbazone group with their anticancer cathepsin L inhibitory activity through the development of a robust 3D QSAR model. Wang et al. [23] presented a comprehensive model with 128 features that allows accurate prediction of allergenic proteins. They showed the value of the Maximum Relevance Minimum Redundancy (mRMR) method and Incremental Feature Selection (IFS) procedure in feature selection. Lastly, Wang et al. [24] developed a novel approach to automatically generate meaningful annotations for gene sets that are directly tied to relevant articles in literature.

## Conference organization

### 2013 International Conference on Intelligent Biology and Medicine (ICIBM 2013)

(August 11-13, 2013, Nashville, Tennessee, USA)

Our sincerest thanks to the members of our Steering, Program, Publication, Workshop/Tutorial, Award, Publicity, Trainee, and Local Organization committees, as well as our numerous reviewers and volunteers, for the countless hours and energy spent to make ICIBM 2013 a success! We could not have accomplished so much without the dedication of each and every person that contributed to this conference.

**Sponsors **National Science Foundation, Vanderbilt University (VU), Vanderbilt Center for Quantitative Sciences, Bioinformatics Resource Center at Vanderbilt-Ingram Cancer Center, International Society of Intelligent Biological Medicine, University of Texas at San Antonio, Shanghai Center for Bioinformation Technology, China, Shanghai Institute for BioMedicine, China, and Shanghai Jiao Tong University, China.

**Partners **Vanderbilt University (Center for Quantitative Sciences, Bioinformatics Resource Center), Meharry Medical College, and Tennessee State University.

**General Chairs **Zhongming Zhao (Vanderbilt University) and Yu Shyr (Vanderbilt University).

**Steering Committee **Chair: Zhongming Zhao (Vanderbilt University). Members: Kevin Johnson (Vanderbilt University), Tony Hu (Drexel University), Jason Moore (Dartmouth College), Limsoon Wong (National University of Singapore), Dong Xu (University of Missouri - Columbia), Ying Xu (University of Georgia).

**Program Committee **Chair: Bing Zhang (Vanderbilt University), Co-Chair: Jason E. McDermott (Pacific Northwest National Laboratory), Members: Kristen Anton (Dartmouth College), William S. Bush (Vanderbilt University), Jake Chen (Purdue University), Xue-Wen Chen (Wayne State University), Juan Cui (University of Georgia), Qinghua Cui (Peking University), Youping Deng (Rush University Medical Center), Joshua Denny (Vanderbilt University), Jason Ernst (University of California, Los Angeles), Jennifer M. Fettweis (Virginia Commonwealth University), Marcelo Fiszman (National Library of Medicine, National Institutes of Health), Jan Freudenberg (Feinstein Medical Research Institute), Ge Gao (Peking University), Mark Gerstein (Yale University), Chittibabu (Babu) Guda (University of Nebraska Medical Center), Yan Guo (Vanderbilt University), Steve Horvath (University of California, Los Angeles), Weichun Huang, (National Institute of Environmental Health Sciences), Yang Huang (Kaiser Permanente), Yufei Huang (University of Texas, San Antonio), Jenn-Kang Hwang (National Chiao Tung University, Taiwan), Peilin Jia (Vanderbilt University), Yufang Jin (University of Texas, San Antonio), Victor Jin (University of Texas, San Antonio), Sun Kim (Seoul National University, Korea), Judith Klein-Seetharaman (University of Warwick, UK), Dmitry Korkin (University of Missouri - Columbia), K.B. Kulasekera (University of Louisville), Leping Li (National Institute of Environmental Health Sciences), Liao Li (University of Delaware), Honghuang Lin (Boston University), Chunyu Liu (University of Chicago), Hongfang Liu (Georgetown University), Qi Liu (Vanderbilt University), Tianming Liu (University of Georgia), Yulong Liu (Indiana University), Zhandong Liu (Baylor College of Medicine), Zhiyong Lu (NCBI, National Library of Medicine), Xinghua Lu (University of Pittsburgh), Patricio A. Manque (Universidad Mayor, Chile), Ranadip Pal (Texas Tech University), Yonghong Peng (University of Bradford), Horacio Perez-Sanchez (Catholic University of Murcia, Spain), Jiang Qian (Johns Hopkins University), Thomas Rindflesch (National Institutes of Health), Marylyn Ritchie (Penn State University), Bairong Shen (Soochow University, China), Alexander Statnikov (New York University Langone Medical Center), Jingchun Sun (Vanderbilt University), Wing-Kin Sung (National University of Singapore, Singapore), P.S. Thiagarajan (National University of Singapore, Singapore), Manabu Torii (Georgetown University Medical Center), Jun Wan (Johns Hopkins University), Jing Wang (Vanderbilt University), Yufeng Wang (University of Texas at San Antonio), Qingguo Wang (Vanderbilt University), Xiaoyan Wang (University of Connecticut), Yonghui Wu (Vanderbilt University), Junfeng Xia (Anhui University, China), Lu Xie (Shanghai Center for Bioinformation Technology, China), Hua Xu (The University of Texas Health Science Center at Houston), Jianhua Xuan (Virginia Tech), Sungroh Yoon (Seoul National University, Korea), Yanqing Zhang (Georgia State University), Min Zhao (Vanderbilt University), Huiru (Jane) Zheng (University of Ulster), W. Jim Zheng (The University of Texas Health Science Center at Houston), and Dongxiao Zhu (Wayne State University).

**Publication Committee **Chair: Yufei Huang (University of Texas, San Antonio), Co-Chair: Yunlong Liu (Indiana University).

**Workshop/Tutorial Committee **Chair: David Tabb (Vanderbilt University).

**Award Committee **Chair: Hua Xu (The University of Texas Health Science Center at Houston), Co-Chair: Sachin Shetty (Tennessee State University), Member: Siddharth Pratap (Meharry Medical College), Yonghui Wu (The University of Texas Health Science Center at Houston).

**Publicity Committee **Chair: Lang Li (Indiana University), Co-Chair: Lu Xie (Shanghai Center for Bioinformation Technology, China).

**Trainee Committee **Chair: Qingguo Wang (Vanderbilt University), Co-Chair: Mario Flores (University of Texas at San Antonio).

**Local Organization Committee **Chair: Rebecca H. Posey (Vanderbilt University). Members: Jillanne K. Shell (Vanderbilt University), Qi Liu (Vanderbilt University), and Qingguo Wang (Vanderbilt University).

## Competing interests

The authors declare that they have no competing interests.

## References

[B1] 2012 International Conference on Intelligent Biology and Medicine http://bioinfo.mc.vanderbilt.edu/icibm/index.html

[B2] Zhao Z Huang Y Zhang B Shyr Y Xu H Genomics in 2012: challenges and opportunities in the next generation sequencing era BMC Genomics 2012 14 Suppl 8 S1 23281891 10.1186/1471-2164-13-S8-S1 PMC3535713

[B3] Huang Y Zhao Z Xu H Shyr Y Zhang B Advances in systems biology: computational algorithms and applications BMC Syst Biol 2012 14 Suppl 3 S1 10.1186/1752-0509-6-S3-S1 23281622 PMC3524016

[B4] Zhao Z Xu H Zhang B Huang Y Advances in intelligent biology and medicine International journal of computational biology and drug design 2013 14 1-2 1 4 23428469 10.1504/IJCBDD.2013

[B5] 2013 International Conference on Intelligent Biology and Medicine http://bioinfo.mc.vanderbilt.edu/icibm2013

[B6] Udyavar A Hoeksema M Clark J Zou Y Tang Z Li M Chen H Statnikov A Li Z Shyr Y Co-expression network analysis identifies Spleen Tyrosine Kinase (SYK) as an oncogenic driver in small-cell lung cancer BMC Systems Biology 2013 14 Suppl S5 S1 24564859 10.1186/1752-0509-7-S5-S1 PMC4029366

[B7] Yu H Lin C Li Y Zhao Z Dynamic protein interaction modules in human hepatocellular carcinoma progression BMC Systems Biology 2013 14 Suppl S5 S2 24564909 10.1186/1752-0509-7-S5-S2 PMC4029569

[B8] Budd W Seashols S Weaver D Joseph C Zehner Z A Networks Method for Ranking microRNA Dysregulation in Cancer BMC Systems Biology 2013 14 Suppl S5 S3 24564923 10.1186/1752-0509-7-S5-S3 PMC4028974

[B9] Shi X Gu J Chen X Shajahan A Hilakivi-Clarke L Clarke R Xuan J mAPC-GibbsOS: An integrated approach for robust identification of gene regulatory networks BMC Systems Biology 2013 14 Suppl S5 S4 24564939 10.1186/1752-0509-7-S5-S4 PMC4028818

[B10] Ma C Chen H Flores M Huang Y Chen Y BRCA-Monet: A Breast Cancer Specific Drug Treatment Mode-of-Action Network for Treatment Effective Prediction Using Large Scale Microarray Database BMC Systems Biology 2013 14 Suppl S5 S5 24564956 10.1186/1752-0509-7-S5-S5 PMC4029357

[B11] Wu C Gudivada RC Aronow B Jegga A Computational drug repositioning through heterogeneous network clustering BMC Systems Biology 2013 14 Suppl S5 S6 10.1186/1752-0509-7-S5-S6 PMC4029299 24564976

[B12] Liu Z Zhang W Wan Y Allen G Pang K Anderson M Molecular pathway identification using biological network-regularized logistic models BMC Genomics 2013 14 Suppl S8 S7 24564637 10.1186/1471-2164-14-S8-S7 PMC4046566

[B13] Jiang J Cui W Vongsangnak W Hu G Shen B Post genome-wide association studies functional characterization of prostate cancer risk loci BMC Genomics 2013 14 Suppl S8 S9 24564736 10.1186/1471-2164-14-S8-S9 PMC4042239

[B14] Srinivasan S Guda C MetaID: A novel method for identification and quantification of metagenomic samples BMC Genomics 2013 14 Suppl S8 S4 24564518 10.1186/1471-2164-14-S8-S4 PMC4042266

[B15] Liu B Yi J Jin V QChIPat: a quantitative method to identify distinct binding patterns for two biological ChIP-seq samples BMC Genomics 2013 14 Suppl S8 S3 24564479 10.1186/1471-2164-14-S8-S3 PMC4042236

[B16] Guo Y Li C F Y Y S Evaluation of Read Count Based RNAseq Analysis Methods BMC Genomics 2013 14 Suppl S8 S2 24564449 10.1186/1471-2164-14-S8-S2 PMC4092879

[B17] Liu Q Zhao S Su P Shyr Y Gene and isoform expression signatures associated with tumor stage in kidney renal clear cell carcinoma BMC Systems Biology 2013 14 Suppl S5 S7 10.1186/1752-0509-7-S5-S7 PMC4028983 24564989

[B18] Sun H Xing X Li J Chen Y He Y Li W Wei G Chang X Jia J Li Y Identification of Gene Fusions from Human Lung Cancer Mass Spectrometry Data BMC Genomics 2013 14 Suppl S8 S5 24564548 10.1186/1471-2164-14-S8-S5 PMC4042237

[B19] Zhang F Wang M Tran M Drabier R Novel Alternative Splicing Isoform Biomarkers Identification from High-Throughput Plasma Proteomics Profiling of Breast Cancer BMC Systems Biology 2013 14 Suppl S5 S8 24565027 10.1186/1752-0509-7-S5-S8 PMC4028860

[B20] Dai Y Li S Dong X Sun H Li C Liu Z Ding G Li Y The de novo sequence origin of two long non-coding genes from an inter-genic region BMC Genomics 2013 14 Suppl S8 S6 24564579 10.1186/1471-2164-14-S8-S6 PMC4042238

[B21] Chakraborty A Jiang G Boustani M Skaar T Liu Y Li L Simultaneous inferences based on empirical Bayes methods and false discovery rates in eQTL data analysis BMC Genomics 2013 14 Suppl S8 S8 24564682 10.1186/1471-2164-14-S8-S8 PMC4042241

[B22] Tyagi C Grover S Dhanjal JK Goyal S Goyal M Grover A Mechanistic insights into mode of action of novel natural cathepsin L inhibitors BMC Genomics 2013 14 Suppl S8 S10 24564425 10.1186/1471-2164-14-S8-S10 PMC4042235

[B23] Wang J Zhang D Li J PREAL: Prediction of Allergen Protein by maximum Relevance Minimum Redundancy (mRMR) Feature Selection BMC Systems Biology 2013 14 Suppl S5 S9 24565053 10.1186/1752-0509-7-S5-S9 PMC4029432

[B24] Wang V Xi L Enayetallah A Fauman E Ziemek D GeneTopics - Interpretation of Gene Sets via Literature-driven Topic Models BMC Systems Biology 2013 14 Suppl S5 S10 24564875 10.1186/1752-0509-7-S5-S10 PMC4029197

